# Digital lifestyle treatment improves long-term metabolic control in type 2 diabetes with different effects in pathophysiological and genetic subgroups

**DOI:** 10.1038/s41746-023-00946-0

**Published:** 2023-10-26

**Authors:** Vishal A. Salunkhe, Neha Sinha, Emma Ahlqvist, Rashmi B. Prasad, Svetlana Johansson, Birgitta Abrahamsson, Anders H. Rosengren

**Affiliations:** 1 Department of Neuroscience and Physiology, Sahlgrenska Academy at the University of Gothenburg, Gothenburg, Sweden; 2 Lund University Diabetes Centre, Department of Clinical Sciences Malmö, Lund University, Malmö, Sweden; 3 Region Skåne, Kristianstad, Sweden

**Keywords:** Type 2 diabetes, Patient education

## Abstract

To address the unmet need for scalable solutions for lifestyle treatment, we developed a new digital method to promote behavioral change. Here we report that patients with type-2 diabetes in Sweden (*n* = 331) exposed to the intervention have significantly improved HbA1c during a median follow-up of 1038 days (4 mmol/mol compared with matched controls; *P* = 0.009). This is paralleled by reduced body weight, ameliorated insulin secretion, increased physical activity, and cognitive eating restraints. Participants with high BMI and insulin resistance have an even larger response, as have non-risk allele carriers for the FTO gene. The findings open a new avenue for scalable lifestyle management with sustained efficacy and highlight a previously unrecognized opportunity for digital precision treatment based on genetics and individual pathophysiology. ClinicalTrials.gov NCT04624321.

Lifestyle-related diseases such as type-2 diabetes (T2D) are increasing health problems of large proportions. Structured lifestyle support is a therapeutic cornerstone but is currently offered to less than ten percent of patients because of practical and financial hurdles^1^. Digital tools could enable clinical utility and meet individual preferences in content and timing, but most solutions require coaching, intensified healthcare activities, or user fees, which hinder the broad application. Moreover, there are several critical knowledge gaps^2^.

First, data on long-term efficacy (>6 months) are scarce^2–5^. Second, no previous studies have analyzed how the individual pathophysiology or genetic variants influence the metabolic response. Third, it is unclear how digital tools affect concrete behaviors such as physical activity and physiological measures of insulin resistance and insulin secretion^2–5^.

In view of these knowledge gaps and the large clinical need for scalable lifestyle treatment, we have developed a self-managed digital tool that is based on a new approach combining health information with structured self-reflection to effectively promote behavioral change^6^. While initial analyses of this intervention have focused on subsets of the most frequent users of the tool^6^, we here make a full assessment of patients exposed to the intervention over an extensive follow-up of approximately 3 years. This is important since recent meta-analyses have shown declining metabolic response to lifestyle programs already after 6 months, and international guidelines emphasize that more long-term data are needed^1–5^. Moreover, to address the current gaps in our understanding of which patients benefit most from digital interventions, we also analyzed the influence of pathophysiological and genetic traits on the response and measured the effects on physical activity, eating behavior, and physiological outcomes, which are largely unknown^2–5^.

Patients with dysregulated T2D (HbA1c ≥ 52 mmol/mol) attended regular visits during a median follow-up of 1038 days (interquartile range 443 to 1488; Supplementary Fig. 1, Supplementary Tables 1 and 2). The average HbA1c decreased by 4.2 mmol/mol from baseline to end of follow-up, independent of adherence to the tool, duration of participation, or medication (*n* = 331; Fig. 1). The mean difference between the intervention group and matched controls followed during a corresponding time period was 3.7 mmol/mol (95% CI: −6.5 to −0.9; *P* = 0.009 using an independent *t*-test; Table 1). When adjusting the analyses for changes in glucose-lowering medication during follow-up, the mean difference between the intervention group and controls was 4.7 mmol/mol (95% CI: −7.3 to −2.1). The participants were also compared with patients in a longitudinal cohort who underwent regular metabolic and behavioral assessments during 3 years at the study center without the intervention. While the patients of the two cohorts had similar metabolic control and physical activity at baseline (Supplementary Table 3), the average HbA1c increased by 2 mmol/mol (95% CI: −0.5 to 5.0) over 3 years in the patients who were not exposed to the intervention.

**Fig. 1:**
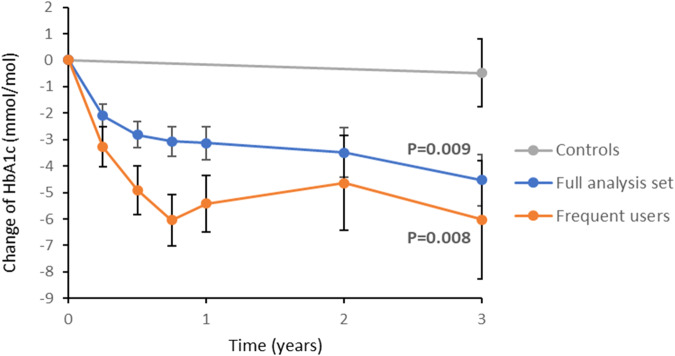
Average changes in glycemic control. Average changes of HbA1c from baseline during follow-up in the full analysis set of the intervention group (*n* = 331; blue lines) and matched controls (grey lines), respectively. Average changes for participants who used the tool more frequently (with a theme completed at least every 14 days, corresponding to the upper quartile of user frequency; *n* = 86; red lines) are also shown. Error bars are s.e.m. *P* values based on independent *t*-tests for comparisons between the full analysis set and controls and between frequent users and controls, respectively, are indicated.

**Table 1:** Descriptive statistics of the full analysis set of study participants and controls^a^.

Characteristic	Intervention group (*n* = 331)	Controls on usual care^b^
Male sex—%	61%	63%
Age—years	63 (9.7)	63 (10.2)
Diabetes duration—years	4.2 (1.4)	3.0 (0.9)
Body mass index^c^	31.1 (5.2)	31.0 (4.8)
Glycated hemoglobin level—mmol/mol	63 (10)	60 (11)
Glucose-lowering medication—%^d^		
None	4.2%	0.0%
Oral only	62.5%	74.1%
Oral and insulin	20.8%	19.6%
Insulin only	6.0%	5.6%
Microvascular complications—%^e^	14.2%	13.7%
Current smokers—%	4.4%	12.3%
Systolic blood pressure (mm Hg)	144 (18)	133 (31)
Diastolic blood pressure (mm Hg)	86 (10)	76 (18)
Total cholesterol (mmol/l)	4.4 (1.2)	4.3 (1.4)
LDL cholesterol (mmol/l)	2.7 (1.0)	2.6 (1.1)
HDL cholesterol (mmol/l)	1.2 (0.3)	1.1 (0.5)
Triglycerides (mmol/l)	2.0 (1.9)	1.6 (0.8)

^a^All participants with at least one measurement of HbA1c after baseline, independent of adherence, duration of participation, or medication were included in the full analysis set (*n* = 331). The table shows demographic and baseline characteristics for the full analysis set and controls, respectively. Data are % or mean (SD).

^b^Controls were matched on a 1:1 ratio exactly on gender and on Mahalanobis distance based on age, body mass index, and glycated hemoglobin level.

^c^The body mass index is the weight in kilograms divided by the square of the height in meters.

^d^Baseline data on glucose-lowering medication were not available from all patients, and percentages do therefore not sum up to 100.

^e^Known microvascular complications, including retinopathy, neuropathy, and nephropathy.

Study participants who used the tool more frequently (at least every 14 days, corresponding to the upper quartile of user frequency) had an even larger metabolic response, with an average HbA1c reduction of 5.5 mmol/mol (−7.6 to −3.4 mmol/mol; *n* = 86; Fig. 1).

As adherence to digital interventions can be variable over time^2–5^, we were interested in the influence of usage patterns on the response to the tool. Those who used the tool during the first year of participation but then ceased using it had an average HbA1c reduction of 6.7 mmol/mol from baseline to end of total follow-up (95% CI: −10.8 to −2.5; average follow-up 802 days; *n* = 49), as compared with −4.7 mmol/mol (95% CI: −6.6 to −2.9) in those who had a more evenly distributed usage throughout the study. This suggests that patients who have been exposed to the tool get sustained effects on glycemic control long after they stop using it.

The reduction of HbA1c was paralleled by improved fasting blood glucose, body weight, and insulin resistance, measured as HOMA2-IR. Moreover, insulin secretion (measured as HOMA2-B), which is the major determinant of disease progression^7^, was improved in study participants compared with controls (Supplementary Table 4).

The effects of the intervention on physical activity were also investigated. During the study, participants reported increased physical activity, with an average increase of 663 metabolic minutes per week relative to baseline (95% CI: 186–1140; *P* = 0.008 using paired *t*-test between first and last measure for each individual with a median 354 days between test and re-test; *n* = 86). This corresponds to 973 kcal higher energy expenditure related to physical activity per week (Supplementary Table 4).

Furthermore, eating behavior was studied along three domains: cognitive restraints, uncontrolled eating, and emotional eating^8^. During the study, participants reported improved cognitive restraints, which includes the awareness and ability to impose limitations on food intake (0.15 a.u [95% CI: 0.04–0.25]; *P* = 0.01 using paired *t*-test between first and last measure for each individual; *n* = 64) without any change in the extent of uncontrolled or emotional eating. The reported usability of the tool was, on average, 70.8 (*n* = 96), as assessed by the System Usability Scale (total score ranging from 0 to 100).

Studies from individuals at risk for diabetes have indicated an impact of body mass index (BMI) and insulin resistance on the effect of exercise programs, but how the individual pathophysiology influences the response to lifestyle interventions in patients with manifest T2D is unclear^9^. We observed an association between baseline BMI and change of HbA1c (beta = −0.30 [95% CI: −0.59 to −0.02]; *n* = 330; Supplementary Table 5) as well as between baseline HOMA2-IR and change of HbA1c (beta = −1.57 [95% CI: −2.64 to −0.50]; *n* = 325).

The influence of genetic variants on the response to lifestyle interventions is also largely unknown. Since the baseline BMI of the participants was associated with the glucose-lowering efficacy of the tool, we wanted to investigate the impact of the FTO gene, which has the strongest genetic association with obesity^10^, on the response. The rs1421085 variant of FTO is strongly associated with obesity and has high linkage disequilibrium with several other risk variants^10^. It was therefore analyzed in 150 of the study participants. Interestingly, the response to the intervention was significantly larger in non-risk allele carriers (−7.9 mmol/mol; *n* = 45) compared with heterozygous or homozygous risk allele carriers (−2.3 mmol/mol; *n* = 105). The mean difference between the genetic groups was 5.6 mmol/mol (95% CI: 0.5–10.7). Adjustments for baseline covariates indicated that this difference was mainly attributed to higher average baseline HbA1c (65 mmol/mol) and lower baseline score on cognitive eating restraints (2.2 a.u.) in non-risk carriers than in risk allele carriers (61 mmol/mol and 2.6 a.u., respectively). The differences for rs1421085 were paralleled by data of another consistent risk variant in FTO, rs9939609 (the variant of FTO that was first associated with obesity^11^). Non-risk allele carriers for rs9939609 (*n* = 50) had a 7.3 mmol/mol reduction of HbA1c on average, compared with a 1.9 mmol/mol average reduction in risk allele carriers (*n* = 108; 95% CI: 0.6–10.3 between the groups). This further suggests that carriers of non-risk haplotype for FTO have a greater response to the intervention.

The digital intervention, with its emphasis on self-reflection, enables a new approach to lifestyle management with sustained efficacy. The present findings extend beyond previous research in digital medicine in three main ways. First, the metabolic improvement was sustained during a median follow-up of three years and was of similar magnitude to common glucose-lowering drugs^12,13^, suggesting broad applicability. The average reduction of HbA1c in patients exposed to the tool was 4 mmol/mol compared with controls, independent of adherence.

Second, since long-term adherence to lifestyle intervention programs is often variable^14^, it is of note that participants who ceased using the tool after the first year had sustained reductions of HbA1c during the entire follow-up period, more than a year after their last session. They also sustained improvement of insulin secretion, which is the major determinant of disease deterioration^7^. In this context, it is of interest that the enhancement of secretory function by pharmacological treatment, such as glucagon-like peptide-1 receptor agonists, is lost almost immediately after treatment discontinuation^15^.

Third, as there is considerable interest in personalizing the treatment of lifestyle diseases based on pathophysiological characteristics, it is of relevance that both baseline BMI and baseline insulin resistance were associated with the change of HbA1c, suggesting that obese insulin-resistant individuals benefit particularly from the tool. Moreover, it is currently unknown how genetic risk variants influence the response to lifestyle interventions. The FTO region has the strongest genetic association with obesity, and previous studies have indicated that risk allele carriers for FTO benefit metabolically from healthy behaviors such as physical activity^16^. Accordingly, the present data demonstrate improved HbA1c in risk allele carriers for FTO exposed to the intervention. Interestingly, the results also show that participants who do not carry the risk haplotype have an even larger response to the intervention. This was mainly attributed to higher baseline HbA1c and lower cognitive eating restraints in non-risk compared with risk allele carriers. Multiple genetic variants influence metabolic control^16^, but it is conceivable that individuals with increased genetic risk for obesity are more prone to develop T2D independently of lifestyle, while metabolic control is more related to behavioral factors in non-risk allele carriers, who would consequently benefit more from a lifestyle intervention. It is also of note that previous studies have shown that the risk allele for rs1421085 reduces oxygen consumption and mitochondrial thermogenesis^10^. The present data represent a step to better understand the heterogeneity of treatment response to lifestyle interventions and help identify those who are most likely to have a pronounced effect. As this could have important clinical and cost-effectiveness implications, future studies should further explore how lifestyle treatment can be personalized based on pathophysiological and genetic characteristics.

Participants were followed for approximately 3 years, making this one of the most extensive investigations of a digital lifestyle intervention to date. The provision as a stand-alone support under conditions that were as similar as possible to the everyday contexts of the patients increases the general significance of the results by demonstrating what can be expected in real-life situations over extended time. It also means that the tool can be provided at a very low cost.

The strengths of the study are the extensive follow-up, the longitudinal assessments of behavioral and physiological measures, the analysis of differential effects based on pathophysiology and genetics, and the novel approach for lifestyle treatment provided by this tool. The study also has a number of limitations. All participants had access to the tool and were compared with matched controls. We used this approach to reduce the risk for unbalanced drop-out and consequently skewed comparisons between exposed and unexposed groups over time, as it may be difficult to motivate a randomized control group that declined the intervention (the assignment is necessarily unblinded) to attend regular visits during several years. It has also been shown that individuals randomized to a control group may be affected in unpredictable ways, including changed motivation and expectancy artefacts^17^. In view of those risks, matched controls may enable more stable comparisons. On the other hand, the risk of placebo and selection bias is increased^17^. To explore the extent to which selection bias or placebo may skew the analyses, the participants were also compared with patients in a longitudinal cohort who underwent metabolic and behavioral assessments during three years without the intervention (Supplementary Table 3). These patients had a corresponding self-selection procedure for participation, attended visits at comparable frequency, and interacted with the same study personnel as users of the tool. While the patients of the two cohorts had similar metabolic control, physical activity, social support, and perceived ability to manage diabetes at baseline, the average HbA1c increased by 2 mmol/mol (95% CI: −0.5 to 5.0; Supplementary Table 3) over three years in the patients who were not exposed to the intervention, as compared with an average reduction of 4 mol/mol in users of the tool. This makes it unlikely that selection bias or placebo explains the major fraction of the glycemic improvement. It is also corroborated by the observation that participants who used the tool frequently (at least biweekly) during the long-term follow-up had a pronounced response, suggesting that improved glucose control is associated with exposure to the tool.

In summary, this study showed that patients with type 2 diabetes exposed to the digital tool had improved long-term metabolic control. While previous meta-analyses have shown declining effect of lifestyle programs after 6 months^5,9^, the metabolic improvement in response to the intervention was sustained during the entire follow-up of approximately 3 years. The tool provides a self-reflective approach to lifestyle treatment that has previously been lacking^18^ and does not require additional healthcare resources, which is often a major hurdle for broad utility. It therefore opens an avenue to address the large unmet need for scalable treatment of lifestyle diseases with sustained efficacy.

## Methods

### The intervention

A detailed description of the intervention and its systematic development with patients is described elsewhere^6^. Briefly, it is based on the theoretical foundation of self-affirmation^19^ and motivational interviewing^20^ and implemented as a digital tool to enable broad applicability. The tool is web- and app-based and is used as a stand-alone continuous support without coaches or the requirement of additional healthcare activities.

Self-affirmation theory is based on the observation that people may react defensively when reminded of unhealthy behaviors and therefore reject the information^19^. Self-affirmation theory postulates that perceived threats to one domain (e.g., sedentary behavior) can be managed more effectively by reflecting on strengths in other domains. From that broader perspective, people may regard changes to specific behaviors, such as eating patterns and physical activity, as less threatening to overall self-integrity, leading to a less defensive attitude^19^. The principles of motivational interviewing were included in the tool to promote reflection on ambivalence and commitment to change, which has been shown to increase intrinsic motivation^20^. To enable this without a traditional interviewer, the digital tool contains a large number of questions to stimulate self-reflection. Self-reflection has played an important role in, for example, later forms of cognitive behavioral therapy^20^ but has not been of major focus in diabetes self-management.

The tool is composed of 80 different themes, focusing on diet and exercise but also a range of other areas, including stress management, decision-making, social interactions, loneliness, and the emotional burden of having diabetes.

A theme takes approximately 10–30 min to complete and contains one or more of the following components: self-assessment tests to raise awareness of current behavior, structured methods to promote behavioral change and information on topics of relevance to health and lifestyle-related diseases. Texts are based on international consensus recommendations for lifestyle management^18^. All texts were written by scientists specifically for this tool and underwent careful editing for style, tone, and language by a panel of physicians, nurses, psychologists, journalists, and literary experts, followed by final approval for accuracy and clarity by the principal investigator.

The tool is used as a continuous support in daily life without a finite number of sessions. There is a diary function for bookmarking texts, writing comments, and getting an overview of personal progression. Users receive regular email prompts about their next round. The tool also contains video webinars and recordings with scientists in different health areas. Patients use the tool at their own pace. They can follow a prespecified order of themes or complete them at their own preference. The various themes allow users to see how different areas are connected and how problems in one domain, e.g., unhealthy eating, could be managed by changes in other domains, such as stress coping. Since initial patient interviews during the development phase identified a need to frame diabetes management within a larger perspective^6^, we also include a set of themes covering the different aspects of existential health that the World Health Organization (WHO) has proposed^21^. These themes aim to stimulate questions on overall life context and how it relates to current habits and disease coping.

The tool is maintained and provided via academic institutions (Universities of Gothenburg and Lund, Sweden). It is technically prepared for multiple languages with a language select function and is currently available in English and Swedish. The tool is constructed to enable large scalability and can be implemented as stand-alone support or combined with other lifestyle management activities. It can be easily accessed directly by patients or healthcare providers.

To maintain privacy, healthcare providers are not able to access user data. Technical functions are, however, prepared to enable users to download a report of their personal questions and reflections in case they wish to share all or parts of their activities on the tool with healthcare professionals to facilitate consultation (this functionality was not employed in the present evaluation study).

### Study design

We conducted a clinical study (approved by the regional ethics review committee in Gothenburg; 651/2016) to test the hypothesis that glucose control would improve in patients with type 2 diabetes exposed to the digital tool. In the study, participants were randomized to access the tool or wait for 12 weeks (1:1 ratio). The two groups were then merged to enable all participants to use the tool during an extended open-label period. Previous reports have focused on the early randomized evaluation and initial open-label follow-up of the most frequent users^6^. Here we report the full long-term analysis of participants. The change of HbA1c in study participants from baseline to end of follow-up (up to 31 Dec 2022) was compared with matched controls on usual care who were followed during a corresponding time period. Usual care refers to routine diabetes management and treatment based on the general guidelines from the European Association for the Study of Diabetes and the American Diabetes Association.

Study personnel were instructed to remain neutral at blood sampling visits and not reinforce usage in order to assess the frequency of use and resultant outcomes that can be expected in real-life situations over an extended time without the need for increased healthcare support. Technical problems were referred to a study coordinator, who also responded to requests to clarify content in a general manner without providing personal advice. The study was performed according to the Declaration of Helsinki and Good Clinical Practice. The protocol was approved by the regional Ethics Review committee in Gothenburg and is appended to the paper. All participants provided written informed consent. The tool is provided via academic institutes. The study was conducted by academic investigators, and funders had no role in design, data collection, analysis, or interpretation.

### Enrolment of participants and study procedures

Patients older than 35 years with prior documentation of type 2 diabetes and HbA1c ≥ 52 mmol/mol (standard glycemic treatment target according to current guidelines) were eligible for enrolment. Diabetes mellitus was diagnosed in routine healthcare based on the WHO criteria (fasting plasma glucose ≥ 7.0 mmol/l or 2-h post-load plasma glucose ≥ 11.1 mmol/l or HbA1C ≥ 48 mmol/mol). All participants provided written informed consent. Exclusion criteria were: type 1 diabetes, maturity-onset diabetes of the young, secondary diabetes, other conditions, treatments, or participation in clinical studies that, in the judgment of the investigator, could affect the evaluation.

Participants were recruited via letters sent to patients with a known history of type 2 diabetes in the All New Diabetics in Scania (ANDIS) cohort or by advertisements. Those who met the study criteria attended regular study visits every third month during the first year and thereafter every 6 months. Each visit lasted approximately 20 min and included blood sampling and anthropometric measurements.

The study center was located at Scania University Hospital, Malmö, Sweden. Participants received travel reimbursement but no other financial incentives. Study participants were managed by their ordinary healthcare providers throughout the study.

### Selection of matched controls

Controls were selected from patients with type 2 diabetes in the ANDIS cohort. ANDIS was approved by the regional ethics review committee in Lund (584/2006 and 676/2012) and aims to include all incident cases of diabetes in Scania, which is one of the largest regions in Sweden with 1,200,000 inhabitants in both rural and urban areas and a wide distribution of socioeconomic background. Approximately 25,000 diabetic patients (>90% of the estimated number of eligible cases in the region) are included. Most individuals with type 2 diabetes are managed in primary care. Prospective data on metabolic variables and medication are obtained via the Swedish National Diabetes Registry and clinical registries on drug prescription and laboratory tests.

The controls were matched exactly with study participants on gender and on Mahalanobis distance based on age (at the index date), body mass index (BMI), and HbA1c. The first possible index date for controls was set to be at least 2 years after the diagnosis date. The index date was selected at random among all registrations meeting the requirements for available follow-up time. Selection of the index date and potential controls were done in a Statistical Analysis System (SAS) program. Balance before and after matching was evaluated using the standardized mean difference.

In addition to matched controls, the change of HbA1c in the intervention group was also compared with patients in a cohort of 48 patients with type 2 diabetes and baseline HbA1c ≥ 52 mmol/mol that had semiannual visits at the study center for metabolic and behavioral analyses during three years without exposure to the intervention^6^. These patients were recruited from ANDIS via letters or advertisements by a corresponding recruitment process as the intervention group. They attended visits at similar frequency and interacted with the same study personnel as users of the tool. All patients provided informed consent (approved by the regional ethics review committee in Lund, 2013/84).

### Baseline data

The following baseline data were collected at the first visit: age, sex, length, body weight, level of education, socioeconomic status, time since diabetes diagnosis, and current glucose-lowering treatment.

### Clinical study outcomes

The primary study variable was HbA1c in blood. Secondary variables included body weight, fasting blood glucose, and homeostasis model assessment-2 estimates of insulin resistance (HOMA2-IR) and beta-cell function (HOMA2-B) based on fasting glucose and C-peptide.

Blood samples were taken in the morning (between 7.30 and 10.00). Participants were instructed to be fasted since 10 pm the previous day and to avoid nicotine use the same day and alcohol consumption and strenuous physical activity within 24 hours of the visit. Fasting blood glucose was measured at the study center using a HemoCue Glucose System (HemoCue AB, Sweden). HbA1c was analyzed according to the International Federation of Clinical Chemistry (IFCC) standard by a Capillary 3 TERA Haemoglobin A1c Kit. C-peptide was measured on Cobas (Roche Diagnostics, Mannheim, Germany).

HOMA2-IR and HOMA2-B were calculated based on C-peptide concentrations (which perform better than insulin in individuals with type-2 diabetes) using the HOMA calculator (University of Oxford, Oxford, UK).

### Patient-reported outcomes

Participants completed the International Physical Activity Questionnaire (IPAQ), which assesses intense and moderate physical activity as well as walking during the last 7 days^22^. The questionnaire was completed online at baseline and during follow-up by participants on repeated occasions, and responses were converted to Metabolic Equivalent Task minutes (MET-minutes) per week according to the IPAQ scoring protocol^22^. MET-minute scores are equivalent to kilocalories for a 60-kg person, and the number of kilocalories was computed from MET-minutes as

$${\rm{MET}}-{\rm{minutes}}\times ({\rm{body}}\,{\rm{weight}}\,{\rm{in}}\,{\rm{kilograms}}/60\,{\rm{kilograms}}).$$

Participants also completed the three-factor eating questionnaire, which is a widely used self-assessment scale to assess various types of eating behavior, including uncontrolled eating, emotional eating, and restrictive eating^8^. Study participants completed it online using a four-point Likert scale, and the items were summed and analyzed along three domains: uncontrolled eating, cognitive eating restraints, and emotional eating.

The usability of the tool was assessed by the system usability scale, which contains ten items with a total score ranging from 0 to 100 (with 100 being the highest and scores above 68 considered to indicate good usability)^23,24^.

### Genotyping

The rs1421085 and rs9939609 single nucleotide polymorphisms of the fat mass and obesity-associated (FTO) gene were genotyped from genomic DNA on an ABI 7900 sequencer (Applied Biosystems).

### Statistics

The primary endpoint was the change of HbA1c from baseline to end of follow-up. The tool was provided in addition to current medication (as prescribed by the ordinary physician) and compared with matched controls on standard care. The baseline for study participants was defined as HbA1c before getting access to the tool. The full analysis set in this study includes all participants who have at least one measurement of HbA1c after baseline, independent of adherence, duration of participation, or glucose-lowering medication. Missing data were not imputed. In a supportive analysis, we also adjusted for changes in glucose-lowering medication during follow-up in study participants and matched controls, such that data from the last measurement with unchanged medicines was used in the analysis.

The change in HbA1c was compared between study participants and matched controls (1:1 ratio) by a two-sided independent t-test. We needed 142 participants in each group to have 80% power at alpha=0.05 to detect a significant difference between the groups, assuming that the true treatment effect of the tool is 2 mmol/mol with a standard deviation of 6 mmol/mol for the change of HbA1c. A considerable surplus was recruited to account for potential dropouts.

Secondary endpoints included the change of secondary variables from baseline to end of follow-up between the intervention group and controls. For controls, data on body weight was obtained via ANDIS and the Swedish National Diabetes Registry, and data on fasting glucose, HOMA2-IR, and HOMA2-B were obtained from longitudinal measurements of ANDIS patients at the Clinical Research Center, Malmö, Sweden. The differences between study participants and controls were compared using independent *t*-tests and are presented as averages with 95% confidence intervals.

Changes in physical activity and eating scores between initial and final tests were analyzed in study participants by paired comparisons and presented as a point estimate with a 95% confidence interval.

The association between baseline variables and changes in HbA1c was analyzed using linear regression.

Summary statistics are presented as point estimates with 95% CI. The widths of the intervals have not been adjusted for multiplicity. Statistical analyses were performed using SPSS (v26). The study is registered at ClinicalTrials.gov NCT04624321 (10 Nov 2020).

### Reporting summary

Further information on research design is available in the Nature Research Reporting Summary linked to this article.

### Supplementary information

Supplementary information clean

Reporting Summary

## Data Availability

The data that support the findings of this study are available from the corresponding author upon reasonable request for non-commercial purposes. The trial protocol is appended to the paper.
