# Supplementary material for: Digital lifestyle treatment improves long-term metabolic control in type 2 diabetes with different effects in pathophysiological and genetic subgroups
Source: NPJ Digit Med. 2023 Oct 26;6:199. doi: 10.1038/s41746-023-00946-0 (PMC10603160; doi:10.1038/s41746-023-00946-0)
Supplement: Supplementary file 1 — Supplementary information clean [file 41746_2023_946_MOESM1_ESM.pdf]

Digital lifestyle treatment improves long-term metabolic control in type 2 diabetes with different effects in pathophysiological and genetic subgroups

Supplementary Information

SUPPLEMENTARY FIGURES

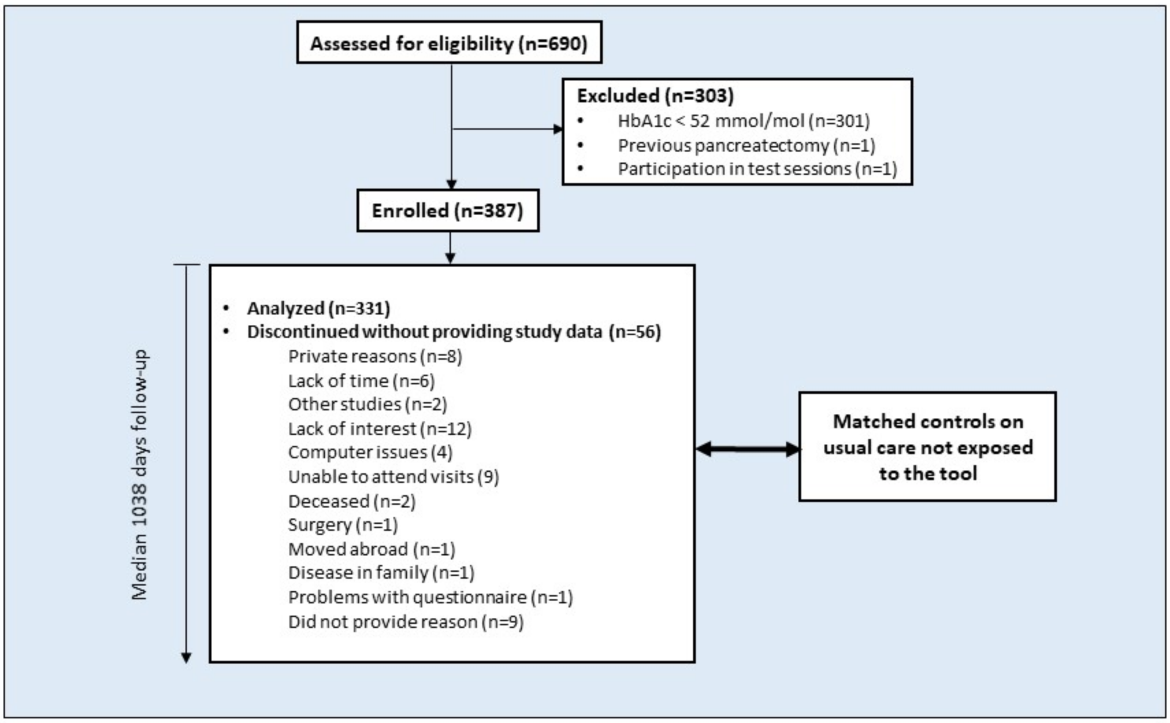

Supplementary Figure 1. Study profile as CONSORT diagram.

A total of 387 individuals were enrolled. Fifty-six participants discontinued after baseline measurements for the long-term follow-up. They had consequently no study data after baseline and could not be analysed. The full analysis set includes all participants who had at least one measurement of HbA1c after baseline, independent of adherence, duration of participation or medication. Participants were followed for a median of 1038 days (interquartile range 443 to 1488; total follow-up time varied between participants, as they were not all recruited simultaneously). The change of HbA1c from baseline was compared between the participants and matched controls on usual care.

SUPPLEMENTARY TABLES

Supplementary Table 1. Demographic and baseline characteristics of the study participants. *

Characteristic	Analysed (n=331)	Discontinued (n=56)	All (n=387)
Male sex – %	61%	68%	62%
Age – years	63 (9.7)	67 (9.9)	63 (9.8)
Diabetes duration – years	4.2 (1.4)	4.1 (1.6)	4.2 (1.4)
Body mass index**	31.1 (5.2)	31.1 (5.3)	31.1 (5.3)
Glycated haemoglobin level – mmol/mol***	62.8 (9.5)	63.8 (9.8)	62.8 (9.4)
Glucose-lowering medication – %			
None	14 (4.2%)	0 (0%)	14 (3.6%)
Oral only	207 (62.5%)	30 (54.5%)	237 (61.4%)
Oral and insulin	69 (20.8%)	10 (18.2%)	79 (20.5%)
Insulin only	20 (6.0%)	8 (14.5%)	28 (7.3%)
Socioeconomic status – %			
Employed	137 (41.4%)	7 (12.7%)	144 (37.3%)
Unemployed	7 (2.1%)	0 (0%)	7 (1.8%)
Retired	137 (41.4%)	19 (34.5%)	156 (40.4%)
Sick-leave > 3 months	14 (4.2%)	0 (0%)	14 (3.6%)
Taking care of own household	3 (0.9%)	0 (0%)	3 (0.8%)
Highest education – no. ****			
Basic level	46 (13.9%)	5 (9.1%)	51 (13.2%)
Medium level	93 (28.1%)	5 (9.1%)	98 (25.4%)
College/University	147 (44.4%)	16 (29.1%)	163 (42.4%)

*All participants with at least one measurement of HbA1c after baseline, independent of adherence, duration of participation or medication were included in the analyses (n=331). Fifty-six participants discontinued after baseline measurements and are reported separately for comparisons. Data are n (%) or mean (SD). Data on socioeconomic status and education were not available from all participants.
**The body mass index is the weight in kilograms divided by the square of the height in meters.
***Glycated haemoglobin level (HbA1c) was analysed according to International Federation of Clinical Chemistry (IFCC) standard.
****Basic level refers to up to 9 years of education; medium level is up 12 years of education.

Supplementary Table 2. Demographic and baseline characteristics of dropouts during follow-up.		
**Characteristic**	**Lost to follow-up (n=49)***	**Remaining participants (n=282)**
Male sex – %	61%	61%
Age – years	64 (9.1)	62 (9.8)
Diabetes duration – years	4.4 (1.3)	4.2 (1.4)
Body mass index**	31.2 (4.8)	31.1 (5.3)
Glycated haemoglobin level – mmol/mol***	63.8 (10.3)	63.0 (10.2)
Glucose-lowering medication – no.		
None	2 (4.4%)	12 (4.5%)
Oral only	31 (67.4%)	176 (66.7%)
Oral and insulin	9 (19.6%)	60 (22.3%)
Insulin only	4 (8.7%)	16 (6.1%)
Socioeconomic status – no.		
Employed	14 (40.0%)	123 (46.8%)
Unemployed	1 (2.9%)	6 (2.3%)
Retired	19 (54.3%)	118 (44.9%)
Sick-leave > 3 months	1 (2.9%)	13 (4.9%)
Taking care of own household	0 (0%)	3 (1.1%)
Highest education – no. ****		
Basic level	5 (14.7%)	41 (16.3%)
Medium level	14 (41.2%)	79 (31.1%)
College/University	15 (44.1%)	132 (52.4%)

*Baseline characteristics of participants lost to follow-up. These participants had <365 days follow-up time and were censored in the analysis such that data up to the last measurement before discontinuation was included. Thus, they were included in the analyses but had shorter follow-up time.

Reasons for discontinuation were: move abroad (n=1), already achieved good results with the tool and feel no need for continuation (n=1), difficulties to attend study visits (n=3), other disease which hinders participation (n=2), or no reason reported (n=42).

Data are n (%) or mean (SD). Data on socioeconomic status and education were not available from all participants.

**The body mass index is the weight in kilograms divided by the square of the height in meters.

***Glycated haemoglobin level (HbA1c) was analysed according to International Federation of Clinical Chemistry (IFCC) standard.

****Basic level refers to up to 9 years of education; medium level is up 12 years of education.

Supplementary Table 3. Baseline characteristics and changes of HbA1c in a patient cohort followed for longitudinal analysis without the intervention compared with the intervention group. *		
**Characteristic**	**Cohort without the intervention (n=48)**	**Intervention group (n=331)**
Male sex – no.	67%	61%
Age – years	65 (6.8)	63 (9.7)
Diabetes duration – years	4.7 (2.9)	4.2 (1.4)
Body mass index**	29.9 (4.5)	31.1 (5.2)
Glycated haemoglobin level – mmol/mol	56.6 (5.0)	63.1 (10.2)
Glucose-lowering medication – no.		
None	6.3%	4.2%
Oral only	79,2%	62.5%
Oral and insulin	12.5%	20.8%
Insulin only	2.1%	6.0%
Socioeconomic status – no.		
Employed	17 (35.4%)	137 (41.4%)
Unemployed	3 (6.3%)	7 (2.1%)
Retired	15 (31.3%)	137 (41.4%)
Sick-leave > 3 months	2 (4.2%)	14 (4.2%)
Taking care of own household	1 (2.1%)	3 (0.9%)
Highest education – no. ****		
Basic level	10 (20.8%)	46 (13.9%)
Medium level	15 (31.3%)	93 (28.1%)
College/University	13 (27.1%)	147 (44.4%)
Baseline physical activity – metabolic minutes per week***	2648 (2111)	2417 (3076)
General health****	2.5 (0.9)	2.8 (1.0)
Social support in managing diabetes*****	5.3 (1.8)	5.8 (1.5)
Self-reported ability to manage diabetes******	5.5 (1.4)	5.4 (1.4)
**Changes during follow-up*******		
Change of glycated haemoglobin level – mmol/mol	2.2 (-0.5 to 5.0)	-4.2 (-5.7 to -2.7)

*A cohort of 48 patients with type 2 diabetes and glycated haemoglobin (HbA1c) ≥ 52 mmol/mol underwent semiannual visits for metabolic and behavioural analyses during a median follow-up of 960 days without exposure to the intervention. Baseline data and changes of glycated haemoglobin (HbA1c) are reported for this cohort as well as for the intervention group. Data are % or mean (SD).
**The body mass index is the weight in kilograms divided by the square of the height in meters.
*** Self-reported data via the International Physical Activity Questionnaire.
****Self-reported general health on a Likert scale from 1 to 4 (where 1 is very good and 4 is very poor)
*****Self-reported social support in managing diabetes on a Likert scale from 1 to 7, where 7 is highest.
******Self-reported ability to manage the challenge posed by diabetes on a Likert scale from 1 to 7, where 7 is highest ability; from the Perceived competence of diabetes scale (Williams GC et al., *Diabetes Care* 21 (1998) 1644-1651).
*******Changes of HbA1c in the longitudinal cohort without intervention and the intervention group, respectively, are reported as point estimates with 95 % confidence intervals.

Supplementary Table 4. Secondary endpoints*	
**Endpoint**	**Mean difference (95% CI)****
Glycated hemoglobin level – mmol/mol	-3.7 (-6.5 to -0.9)
Fasting plasma glucose – mmol/l	-1.5 (-1.9 to -1.1)
Body weight – kg	-2.2 (-4.1 to -0.3)
HOMA2-IR***	-0.35 (-0.55 to -0.15)
HOMA2-B***	22.9 (17.2 to 28.6)

*Changes from baseline to end of follow-up in participants in the intervention group (n=331) compared with controls on usual care.
**Estimated differences of study participants minus controls are presented as means with 95% CI.
***Homeostasis model assessment-2 estimates of insulin resistance (HOMA2-IR) and beta-cell function (HOMA2-B).

Supplementary Table 5. Association between baseline BMI and change of HbA1c.	
**Group of participants based on baseline BMI**	**Beta coefficient from linear model***
All participants (n=330)	-0.30 (95% CI -0.59 to -0.02)
Participants with body mass index < 28 kg/m^2 (n=94)	-0.63 (95% CI -2.2 to 1.0)
Participants with body mass index ≥ 28 kg/m^2 (n=236)	-0.33 (95% CI -0.7 to 0.1)
**Group of participants based on baseline BMI**	**Change of HbA1c (mmol/mol)****
Participants with body mass index < 25 kg/m^2 (n=33)	-1.2 (1.7)
Participants with body mass index 25-29.9 kg/m^2 (n=117)	-3.1 (1.5)
Participants with body mass index 30-34.9 kg/m^2 (n=112)	-4.7 (1.2)
Participants with body mass index 35-39.9 kg/m^2 (n=44)	-7.5 (1.8)
Participants with body mass index ≥ 40 kg/m^2 (n=25)	-5.4 (2.6)

*The association between baseline body mass index (BMI) and change of glycated haemoglobin (HbA1c) was analysed using linear regression with baseline body mass index as the independent variable and change of HbA1c as the dependent variable. The unstandardized beta coefficient from the linear model is reported with 95% confidence intervals (CI). Data are reported for all participants, for those with BMI <28 kg/m^2 and for those with BMI ≥28 kg/m^2, respectively.
**Mean change of HbA1c in participants with different baseline BMI. Data are mean (s.e.m.).
