# Supplementary material for: Digital lifestyle treatment improves long-term metabolic control in type 2 diabetes with different effects in pathophysiological and genetic subgroups
Source: NPJ Digit Med. 2023 Oct 26;6:199. doi: 10.1038/s41746-023-00946-0 (PMC10603160; doi:10.1038/s41746-023-00946-0)
Supplement: Supplementary file 2 — Reporting Summary [file 41746_2023_946_MOESM2_ESM.pdf]

nature portfolio

Corresponding author(s): Anders Rosengren

Last updated by author(s): Sep 30, 2023

Reporting Summary

Nature Portfolio wishes to improve the reproducibility of the work that we publish. This form provides structure for consistency and transparency in reporting. For further information on Nature Portfolio policies, see our Editorial Policies and the Editorial Policy Checklist.

Statistics

For all statistical analyses, confirm that the following items are present in the figure legend, table legend, main text, or Methods section.

n/a	Confirmed	
☐	☒	The exact sample size (*n*) for each experimental group/condition, given as a discrete number and unit of measurement
☐	☒	A statement on whether measurements were taken from distinct samples or whether the same sample was measured repeatedly
☐	☒	The statistical test(s) used AND whether they are one- or two-sided<br>*Only common tests should be described solely by name; describe more complex techniques in the Methods section.*
☐	☒	A description of all covariates tested
☐	☒	A description of any assumptions or corrections, such as tests of normality and adjustment for multiple comparisons
☐	☒	A full description of the statistical parameters including central tendency (e.g. means) or other basic estimates (e.g. regression coefficient) AND variation (e.g. standard deviation) or associated estimates of uncertainty (e.g. confidence intervals)
☐	☒	For null hypothesis testing, the test statistic (e.g. *F*, *t*, *r*) with confidence intervals, effect sizes, degrees of freedom and *P* value noted<br>*Give P values as exact values whenever suitable.*
☒	☐	For Bayesian analysis, information on the choice of priors and Markov chain Monte Carlo settings
☒	☐	For hierarchical and complex designs, identification of the appropriate level for tests and full reporting of outcomes
☒	☐	Estimates of effect sizes (e.g. Cohen's *d*, Pearson's *r*), indicating how they were calculated

Our web collection on statistics for biologists contains articles on many of the points above.

Software and code

Policy information about availability of computer code

Data collection	Data were collected using the treatment tool, which is based on Drupal ver. 8.
Data analysis	Statistical analyses were performed using SPSS (v26).

For manuscripts utilizing custom algorithms or software that are central to the research but not yet described in published literature, software must be made available to editors and reviewers. We strongly encourage code deposition in a community repository (e.g. GitHub). See the Nature Portfolio guidelines for submitting code & software for further information.

Data

Policy information about availability of data

All manuscripts must include a data availability statement. This statement should provide the following information, where applicable:

- Accession codes, unique identifiers, or web links for publicly available datasets
- A description of any restrictions on data availability
- For clinical datasets or third party data, please ensure that the statement adheres to our policy

Data requests should be submitted to the corresponding author for consideration. Access to anonymized data may be granted following review. The trial protocol is appended to the article.

Research involving human participants, their data, or biological material

Policy information about studies with human participants or human data. See also policy information about sex, gender (identity/presentation), and sexual orientation and race, ethnicity and racism.

Reporting on sex and gender	The study enrolled both women and men, and the results are applicable to both women and men. Gender was determined by self-report and the social security number (no discrepancies).
Reporting on race, ethnicity, or other socially relevant groupings	The study includes participants living in southern Sweden in both rural and urban areas and a wide distribution of socioeconomic background.
Population characteristics	Participants had type 2 diabetes and were 35 years or above
Recruitment	Participants were recruited via letters or advertisements and they responded to invitation by self-selection.
Ethics oversight	Swedish Ethical Review Authority (2020-01353)

Note that full information on the approval of the study protocol must also be provided in the manuscript.

Field-specific reporting

Please select the one below that is the best fit for your research. If you are not sure, read the appropriate sections before making your selection.

☒ Life sciences ☐ Behavioural & social sciences ☐ Ecological, evolutionary & environmental sciences

For a reference copy of the document with all sections, see nature.com/documents/nr-reporting-summary-flat.pdf

Life sciences study design

All studies must disclose on these points even when the disclosure is negative.

Sample size	The change of HbA1c was compared between study participants and matched controls (1:1 ratio) by a two-sided independent t-test. We needed 142 participants in each group to have 80% power at alpha=0.05 to detect a significant difference between the groups, assuming that the true treatment effect of the tool is 2 mmol/mol with a standard deviation of 6 mmol/mol for the change of HbA1c. A considerable surplus was recruited to account for potential dropouts.
Data exclusions	The full analysis set in this study includes all participants who have at least one measurement of HbA1c after baseline, independent of adherence, duration of participation or glucose-lowering medication. Missing data were not imputed.
Replication	Findings were consistent with initial studies of the tool (Dwibedi et al., npj Digital Medicine 2022). The secondary variable outcomes also support the primary outcomes, showing broad metabolic improvements, including body weight, insulin resistance and insulin secretion.
Randomization	The intervention group was compared with matched controls.
Blinding	The study is open-label since treatment was based on a digital tool where placebo was not feasible.

Reporting for specific materials, systems and methods

We require information from authors about some types of materials, experimental systems and methods used in many studies. Here, indicate whether each material, system or method listed is relevant to your study. If you are not sure if a list item applies to your research, read the appropriate section before selecting a response.

Materials & experimental systems

n/a	Involved in the study	
☒	☐	Antibodies
☒	☐	Eukaryotic cell lines
☒	☐	Palaeontology and archaeology
☒	☐	Animals and other organisms
☐	☒	Clinical data
☐	☒	Dual use research of concern
☒	☐	Plants

Methods

n/a	Involved in the study	
☒	☐	ChIP-seq
☒	☐	Flow cytometry
☒	☐	MRI-based neuroimaging

Clinical data

Policy information about clinical studies

All manuscripts should comply with the ICMJE guidelines for publication of clinical research and a completed CONSORT checklist must be included with all submissions.

Clinical trial registration	ClinicalTrials.gov identifier: NCT04624321
Study protocol	The study protocol is appended with the paper.
Data collection	Participants attended study visits in Malmö, Sweden, every 6 months for blood sampling and physical examination but received no counselling or lifestyle advice from the study personnel. Technical problems were referred to a study coordinator.
Outcomes	The primary study variable was HbA1c in blood. Secondary variables included body weight, fasting blood glucose, and homeostasis model assessment-2 estimates of insulin resistance (HOMA2-IR) and beta-cell function (HOMA2-B) based on fasting glucose and C-peptide.
